# Preclinical Studies of a Novel Human PD-1 B-Cell Peptide Cancer Vaccine PD1-Vaxx From BALB/c Mice to Beagle Dogs and to Non-Human Primates (Cynomolgus Monkeys)

**DOI:** 10.3389/fonc.2022.826566

**Published:** 2022-05-13

**Authors:** Linlin Guo, Jay Overholser, Anthony J. Good, Nicholas J. Ede, Pravin T. P. Kaumaya

**Affiliations:** ^1^ Department of Obstetrics & Gynecology, The Ohio State University Wexner Medical Center, Columbus, OH, United States; ^2^ Imugene Limited, Sydney, NSW, Australia; ^3^ James Comprehensive Cancer Center, Columbus, OH, United States

**Keywords:** cancer vaccine, peptide B-cell epitope vaccine, immunotherapy, checkpoint inhibitor PD-1, non-human primates, phase 1 human clinical trial, NSCLC

## Abstract

Immunotherapy with monoclonal antibodies to checkpoint inhibitors against the PD-1/PD-L1 signaling pathway is a landmark achievement in cancer therapy. Some anti-PD-1 inhibitors such as nivolumab and pembrolizumab have shown clinical success, in a percentage of patients with prolonged survival rates. However, adverse effects accompany these benefits. In this case, strategies with lower toxicity and increased specificity are urgently required. Cancer vaccines have the ability to stimulate the native immune system and in particular, an engineered B-cell epitope can elicit high-affinity polyclonal antibodies with similar efficacy to PD-1 monoclonal antibodies in murine animal models. We have previously designed and synthesized a unique B-cell vaccine, PD1-Vaxx [MVF-PD-1(92-110)], and we have tested the immunogenicity and antitumor properties in CT26 colon cancer BALB/c syngeneic mice model. This manuscript provides results from comprehensive preclinical pharmacology studies encompassing primary and secondary pharmacodynamics, biodistribution, and safety studies. The results from these preclinical studies support the use of PD1-Vaxx in a first-in-human clinical trial in patients with non-small cell lung cancer (NSCLC). A phase I trial in patients with NSCLC has commenced.

## 1 Introduction

Currently, cancer is a global health problem and millions of people are diagnosed with cancer every year in the United States and worldwide (1, 2). Conventional therapies include radiation, chemotherapy, and targeted therapies, and the latest anti-tumor strategies include cancer vaccines and checkpoint blockade. In 2018, the Nobel Prize in Physiology or Medicine was awarded to Tasuku Honjo and James P. Allison (3) for their contribution in discovering cancer therapy by inhibiting programmed cell death protein 1 (PD-1) and cytotoxic T-lymphocyte antigen 4 (CTLA-4), respectively. This discovery opened a new area of cancer immunotherapy by inhibiting related checkpoints.

Tasuku Honjo’s group discovered PD-1 in 1992 (4) and identified programed cell death ligand 1 (PD-L1) as PD-1 ligand in 2000 (5). One year after that, programmed cell death ligand 2 (PD-L2) was discovered and the expression on tumor cell lines as the second PD-1 ligand (6). Both PD-1 ligands PD-L1 and PD-L2 belong to the B7 family members. The expression of PD-L1 can be found on almost all murine tumor cell lines (7, 8), while the expression of PD-L2 seems only limited in some certain type of tumor cell lines (5); all these discoveries are based on the blockade of the PD-1/PD-L1 signaling pathway. The inhibition of immune checkpoint blockade has revolutionized the therapies for many cancer patients (9, 10). All the current FDA (US Food and Drug Administration)-approved cancer therapies targeting either PD-1 or PD-L1, such as Optivo^®^ (nivolumab), Keytruda^®^ (pembrolizumab), pidilizumab, and Libtayo^®^ (cemiplimab) against PD-1, and Bavencio^®^ (avelumab), Imfinzi^®^ (durvalumab), and Tecentriq^®^ (atezolizumab) against PD-L1, are associated with remarkable response rates in various cancers and have revolutionized cancer treatment.

The success of these drugs are often accompanied by adverse side effects (11–14), and only a subset of patients (10%–30%) respond to monotherapy due to the development of primary and secondary resistance.

Compared with all other cancer therapeutic strategies or immunotherapeutic approaches, cancer peptide-based vaccines might provide unique and effective options for active immunotherapy that induce specific anti-tumor immune responses (15–19). Additionally, peptide vaccines have low toxicity, are offered at a lower cost to patients, and are relatively easy to manufacture (20, 21) with the potential to be a treatment option against cancer in the near future. With relatively limited toxicity and limited serious adverse events, cancer vaccines have the potential to provide promising and effective tumor therapy. In 2010, FDA approved the first cancer vaccine for the therapy of cancer (22), heralding a new era in immuno-oncology.

We have recently developed an anti-PD-1 B-cell epitope called PD1-Vaxx using an active immunization approach to treat participants with tumors that overexpress PD-L1 (23). The hypothesis is that a polyclonal-induced B-cell antibody response will be more effective or as effective with improved safety over the current monoclonal antibody therapy. The expected production of B-cell-derived polyclonal antibodies should produce a durable antibody response and a potentially continuous anti-tumor effect.

An appropriate immunization schedule and selection of an effective adjuvant play a pivotal role in cancer vaccine strategies. In order to simplify and accelerate vaccination protocols and to confirm which immunization schedules can elicit high immunogenicity responses in animals, we investigated whether there is a difference between 2-week interval and 3-week interval immunization strategies, to determine the best schedule and adjuvant given that clinical application requires the fastest attainable immune activation, especially when targeting the treatment of rapidly progressing tumors such as NSCLC.

Additionally, we investigated which adjuvant, Montanide ISA 720 or Montanide ISA 51, created the best induction of an effective immune response with enhanced immunogenicity.

This manuscript summarizes the immunogenicity and antitumor activity and nonclinical pharmacology, pharmacokinetic, and toxicology studies conducted on PD1-Vaxx, which formed the basis for translation from experimental application in animals to clinical application as a potential human immunotherapy. Based on these nonclinical results, PD1-Vaxx has acceptable pharmacology, immunogenicity, and nonclinical safety (safety pharmacology, pharmacokinetics, and toxicology) profiles to justify initiating clinical trials with PD1-Vaxx as monotherapy in NSCLC patients who have been shown to overexpress PD-L1.

## 2 Experimental Methods and Materials

### 2.1 Peptide Synthesis

As previously described (23), we synthesized a novel MVF-PD-1(92-110) peptide vaccine. Briefly, peptide synthesis was performed using 9600 Milligen/Biosearch solid-phase peptide synthesizer (Millipore, Bedford, MA, USA) using Fmoc/t-Butyl chemistry and PyBOP/HOBT coupling reagents on either CLEAR amide resin or CLEAR acid resin (Peptides International, Louisville, KY, USA). All MVF derived chimeric peptide vaccines were co-linearly synthesized with a “*promiscuous*“ Th cell epitope derived from the measles virus fusion protein (MVF; residues 288–302, KLLSLIKGVIVHRLEGVE) using a four-residue linker Glycine-Proline-Serine-Leucine (GPSL). Peptides were cleaved from the resin using cleavage reagent R (TFA)/thioanisole/EDT/anisole (90/5/3/2), and crude peptides were purified by semi-preparative (C-4 or C-18 Vydac columns) reversed-phase High-performance liquid chromatography (HPLC) (Waters, Bedford, MA, USA) and characterized by MALDI (matrix-assisted laser desorption ionization) mass spectroscopy at the CCIC (Campus Chemical Instrumentation Center, The Ohio State University, Columbus, OH, USA). All fractions were analyzed on analytical RP-HPLC and characterized by MALDI. The RP-HPLC fractions showing the same mass spectrum peak were pooled together and lyophilized.

### 2.2 Animals: BALB/c Mice, Beagle Dogs, Cynomolgus Monkeys

As in previous studies, all experiments were performed in accordance with the US Public Health Service Policy on Humane Care and Use of Laboratory Animals and approved by the Ohio State University Institutional Animals Care and Use Committee and detailed in the accepted protocol (2009A0013-R2:2/5/2015-2/5/2018; 2009A0013-R3:1/18/2018-1/18/2021; 2009A0013-R4 and 11/9/2021-11/9/2024). BALB/c mice, beagle dogs, and non-human primate cynomolgus monkeys were used in this study supported by Imugene Limited Australia; the animals were purchased from Charles River Laboratories (Wilmington, MA, USA), and the cynomolgus monkey experiments were contracted and carried out at the Charles River Laboratories Ashland, LLC, United States.

### 2.3 Procedures

#### 2.3.1 Immunization Procedure: Beagle Dog

In brief, the beagle dogs were purchased from Covance Research Products (Cumberland, VA, USA) and were housed in the Ohio State University Veterinarian Facility Institutional Animal Care Facility. The dogs were housed at 19°C−24°C room temperature with approximately 70% humidity, and 12 h light and 12 h dark–light cycle. The dogs’ body weights were at least 7.5 kg when they received the first immunization. There were four dogs in the current study to test PD1-Vaxx, negative control, low dose 1.0 mg, medium dose 1.5 mg, and high dose 3.0 mg, mixed with ISA 720 and nor-MDP. The dogs received a total of 4 doses, immunization was done at the rear thigh with two sites of injections, the bleed was collected as indicated time points to monitor the antibody titers, and the body weight of each dog was monitored closely (see **Figures 3B, C** for detail).

#### 2.3.2 Immunization Procedure: Non-Human Primate

The non-human primate studies were carried out by the Charles River Laboratories (Ashland, OH USA). All the cynomolgus monkeys (*Macaca fascicularis* of Chinese origin) were obtained from Charles River Laboratories (Houston, TX, USA). In brief, the animals were approximately 2–4 years old and at the body weight between 2.2 and 3.1 kg at the initial dosing. The animals were kept at the temperature between 19°C and 24°C, humidity 30%–70%, and with 12 h light and 12 h dark light cycle. The Good manufacturing practice (GMP)-manufactured vaccine was used in the non-human primate study. The dosing formulations [IMU-201 (PD1-Vaxx) with ISA 720 VG vehicle], 50%:50% emulsion with a total volume of 1 ml per dose, vehicle control mixed with sterile water were prepared at appropriate concentrations to meet the dose requirements. Similar to mouse and dog dosing preparation, after the emulsion was prepared, a drop test was performed on each time of dosing to confirm that the emulsion had been prepared properly. If the drops of formulation did not mix with water in the beaker at the room temperature, this indicated that the antigen in oil emulsion had been properly prepared. The dose volume was split approximately equally across two different sites; 0.5 ml of the dose was intramuscularly (IM) injected into the right posterior lateral thigh, the left dosing was injected into the left site. All the animals’ body weights were monitored weekly after receiving. After each immunization, the animal body temperature was tested 6 and 24 h post each immunization. Each of the individual monkeys received 4 doses of the vaccine with a 2-week interval during the study (except half the number of monkeys removed out of the study at day 43 did not receive the last immunization). The animal bleed were collected before each time of immunization to monitor the antibody titers. There were two sets of non-human primate studies conducted. The first set of monkey study No.00213515 had 24 monkeys, 12 males, and 12 females divided into 4 groups: vehicle control, low dose 0.5 mg, medium dose 2 mg, and high dose 5 mg. There were 16 monkeys (8 males and 8 females) in the second set of monkey study No. 00213519. The monkeys in the four groups were vaccinated as follows: vehicle control, low dose 25 μg, medium dose 100 μg, and high dose 250 μg.

### 2.4 Cancer Cell Line and *In Vivo* Study of CT26/Tumor Model on BALB/c Mice

The peptide vaccine MVF-PD-1(92-110) was dissolved in sterile water and mixed with either ISA 720 or ISA 51 (1:1) ratio. We utilized the well-established murine colon carcinoma cell line CT26 tumor model in syngeneic BALB/c mice to evaluate the effects of vaccination previously described (23). Briefly, 6–8-week-old female BALB/c mice were immunized with the peptide vaccine, and 2 weeks after the third immunization, the mice were challenged with 1×10^5^ CT26 colon cancer cells by subcutaneous injection (SC). The CT26 cancer cell line was maintained in the DMEM medium with 10% FBS 5% CO_2_ at 37˚ C. Phosphate-buffered saline (PBS)-treated mice were set as negative control, while the positive control group mice were treated with an anti-mouse PD-1 monoclonal antibody (clone 29F.1A12; Bio X Cell, West Lebanon, NH, USA) with 200 μg/dose per mouse. Blood was collected biweekly after the primary immunization until 2 weeks after the third immunization. Tumor growth was monitored daily, especially 7 days after the tumor challenge. The mice were euthanized at the end of treatment, and the tumors were extracted and weighed.

The mice in PBS and mAb (29F.1A12) groups, n = 10; group 1 to group 7, n = 8, group 8 n = 7 (one mouse was lost due to a non-immunization related reason before the challenge).

### 2.5 Enzyme-Linked Immunosorbent Assay and Human Recombinant Protein Activity Test

Enzyme-linked immunosorbent assay (ELISA) was performed per our lab standard procedure. Briefly, a 96-well plate was coated with 100 μl of MVF-PD-1(92-110) peptide as an antigen at 2 μg/ml in phosphate-buffered saline (PBS; Research Products International, Mt Prospect, IL, USA, CAS No. 7647-145) overnight at 4°C. Nonspecific binding sites were blocked for 1 h with 200 μl of PBS with 1% BSA (bovine serum albumin; Thermo Fisher Scientific, Waltham, WA, USA, BP9703-100), and the plate was washed with a washing buffer (PBS diluted 0.05% Tween 1% horse serum). Vaccine antibodies in the blocking buffer were added to the antigen-coated plate in duplicate wells, serially diluted 1:2 in the blocking buffer, and incubated for 2 h at room temperature. After washing the plates, 100 μl of 1:500 goat anti-rabbit IgG conjugated to horseradish peroxidase (Invitrogen, Waltham, MA, USA, REF:31430) was added to each well and incubated for 1 h at room temperature. After washing, the antibody was detected using 50 μl of 0.15% H_2_O_2_ in 24 mM of citric acid and 5 mM of the sodium phosphate buffer (pH5.2) with 0.5 mg/ml 2, 2’-aminobis (3-ethylbenzthiazole- 6-sulfonic acid, ABTS; Sigma, St. Louis, MO, USA) as the chromophore. Color development proceeded for 10 min, and the reaction was stopped with 25 μl of 1% SDS (sodium dodecyl sulfate; Thermo Scientific, Waltham, WA, USA, Prod#28312). Absorbance was read at 415 nm using an ELISA microplate reader (SPECTRAmax PLUS384; Molecular Devices, San Jose, CA, USA). The highest dilution at the cutoff absorbance 0.2 were determined as the antibody titer.

For the detection of PD-1 human recombinant protein activity, the human recombinant protein PD-1 (PD1-H5221, HIS tag; ACROBiosystems, Newark, DE, USA) 100 μl at the concentration of 10 μl/ml were coated on the 96 wells plate as an antigen. The following procedures were performed as the description of ELISA.

### 2.6 Antibody Isotyping Assay

The experimental assay was carried out by following the manufacturer instruction and lab protocol. The mouse antibody isotypes (i.e., IgA, IgM, IgG1, IgG2a, IgG2b, and IgG3) were determined using the Mouse Typer isotyping kit (BIO-RAD, Hercules, CA, USA, Cat. #172-1722055). Briefly, the wells of a 96-well assay plate (COSTAR, Washington, D.C., USA, REF#2797) were coated with 100 μl of 2 μg/ml peptide antigen in double-distilled water (ddH_2_O) and incubated at 4°C overnight. The plate was washed with a washing buffer (0.05% Tween-20 and 1% horse sera in PBS). The plate was blocked with 1% BSA in PBS at room temperature for 1 h. Approximately 100 μl of diluted sera was added to each well. The dilutions of each sera samples were determined by the ELISA titers shown in the absorbance of 0.4 or higher after subtracting the background. After washing the wells, 100 μl of ready-to-use rabbit anti-mouse subclasses antibodies were added to each well, respectively, and incubated at room temperature for 2 h. The wells were washed again; 100 μl (1/3,000 dilution of goat anti-rabbit conjugated to the HRP antibody) (BIO-RAD, Hercules, CA, USA, Cat. #172-1019) was added to each well and incubated for 1 h at room temperature in dark room. The plate received a final wash, and 50 μl of the prepared substrate solution was added to each well (BIO-RAD, Hercules, CA, USA, Cat. #1721064). The reaction was stopped with 25 μl of a 5% SDS stopping buffer. Absorbance at 415 nm was determined using an ELISA plate reader. The similar procedure was performed on the analysis of canine antibody isotypes (i.e., IgA, IgM, IgG1, IgG2).

### 2.7 Statistical Analysis

The mice challenged with CT26 tumor cells were monitored at least twice per week and tumor sizes were measured by calipers. The formula, volume (LWW) = (length × width × width)/2, was used to calculate tumor volumes. All values are shown as means ± standard deviation. Data statistical analysis was performed by GraphPad Prism 8.1.2 (GraphPad Software, Inc. San Diego, CA, USA) and the indicated statistical analysis. The percentage of tumor growth inhibition (%TGI) was defined as the difference between the median tumor volume (MTV) of the treatment group with the PBS control group, and the value was calculated by the following formula: %TGI=100*(MTV control − MTV test)/MTV control. The percentage of complete response (CR) was defined as the tumor volume equal to or less than 50 mm^3^ for at least 3 consecutive measurements during the study. The log-rank (Mantel−Cox) test was used to compare the percentage of CR in multiple groups. Student’s t-test was used to analyze the difference between the two groups. One-way analysis of variance (one-way ANOVA) followed by the Tukey’s multiple comparison test were used to compare data in multiple groups or data between groups in multiple groups, and the two-way ANOVA was used to analyze the whole curve comparison. The log-rank (Mantel−Cox) test was use to compare the survival curves. A *p*-value or an adjusted *p*-value less than 0.05 was accepted as a statistically significant difference.

## 3 Results

### 3.1 Selection, Design, and Characterization of Peptide Epitopes for Human PD-1

The selection of candidate B-cell epitopes expressed on the surface of PD-1 was accomplished by a computer-aided analysis using six correlates of antigenicity. A potential epitope was selected for further investigation as described in Kaumaya et al. (23).

^1^PPTFSPALL^11^VVTEGDNATF^21^TCSFSNTSES^31^ FVLNWYRMSP^40^

^41^SNQTD45KLAAF^51^PEDRSQPGQD ^61^CRFRVTQLPN ^71^GRDFHMSVVR^80^

^81^ARRNDSGTYL^91^CGAISLAPKL^101^QIKESLRAEL^111^RVTERRAEVP ^121^TAHPSPSP

The PD-1 amino acids 92-110 (^92^GAISLAPKL^101^QIKESLRAEL^110^) chosen for evaluation were selected based on the crystal structure of PD-1:PD-L1. This PD-1 sequence was synthesized as a chimeric construct with a promiscuous T-cell helper epitope derived from the measles virus fusion protein (MVF, amino acids 288-302). The specificity of the selected PD-1 peptide was determined by surface plasmon resonance spectroscopy. The results from binding affinity studies using human PD-L1 and nivolumab (an approved human IgG4 monoclonal antibody that blocks PD-1) showed that MVF PD-1 (92-110), which includes the PD-1 amino acids in APi2568, was able to recognize and bind both rhPD-L1 and nivolumab and thus could act as an inhibitor of PD-1:PD-L1.

#### 3.1.1 GMP Peptide Manufacture

The drug substance (called APi2568) is a 41-amino acid peptide [cGMP batch manufactured by Ambiopharm Inc (North Augusta, GA, USA) and sterile-filled and lyophilized and finished by the University of Iowa Pharmaceuticals Inc. (Iowa City, IA, United States)]. The peptide comprises a B-cell epitope (amino acids 92-110 from the PD-1 receptor) linked to a promiscuous T-cell epitope (amino acid residues 288−302 from measles virus fusion protein) *via* a 4-amino acid linker (Gly-Pro-Ser-Leu).

APi2568 [MVF-PD-1(92-110)] is combined with water for injection to form the aqueous-phase vaccine and drug product called IMU-201. PD1-Vaxx is the water-in-oil emulsion formulation for IM injection prepared with IMU-201 and an adjuvant, Montanide ISA 720 VG.

### 3.2 Pharmacology and Immunogenicity

#### 3.2.1 Assessment of Immunogenicity, Isotype Distribution, Immune Stimulant nor-MDP, ISA 720 Versus ISA 51 and Vaccination Schedules (2v3 Weeks) in BALB/c Mice Challenged With CT26 Colon Carcinoma Cell Lines

**Figure 1:**
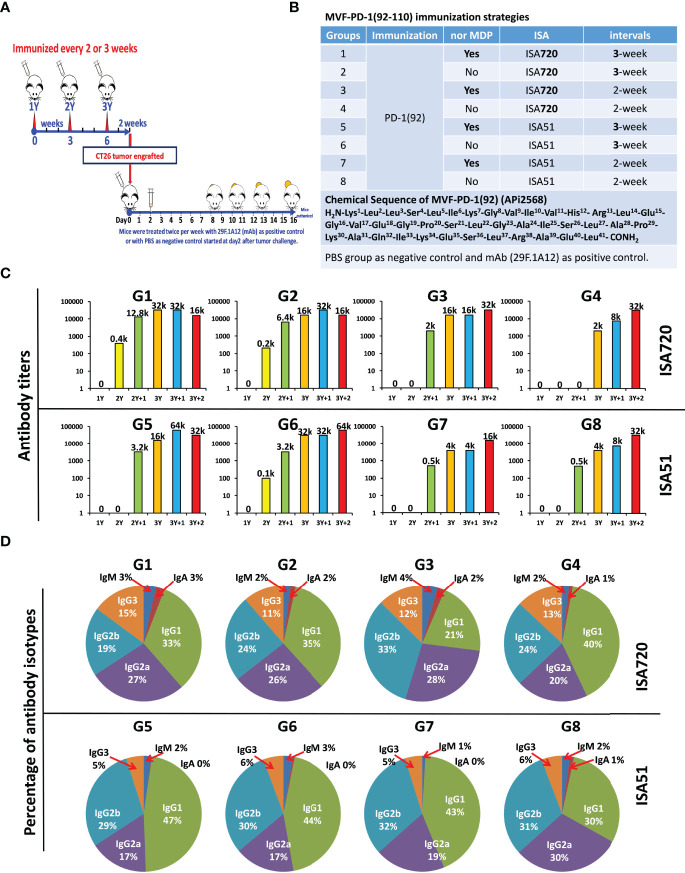
Immunogenicity and antibody isotypes of CT26 BALB/c mice model. **(A)** Approximately 6–8 weeks old female BALB/c mice were vaccinated with MVF-PD-1(92-110) for every 2- or 3-week interval. Peptide cancer vaccine 100 μg mixed with ISA 720 (1:1) or ISA 51 (1:1) or nor-MDP 33 μg were used per mice. Mice were boosted with the designed doses for every 2- or 3-week intervals. Blood was collected weekly for monitoring antibody titers. After 2 weeks of the third-time immunization (3Y), mice were challenged with CT26 colon carcinoma cells for 1 × 10^5^ cells per mice. The tumor growth was checked daily, especially 7 days of post-challenging, and the tumor size was measured with a caliper. For the positive control group, we treated the mice with anti-mouse PD-1 antibody (clone 29F.1A12) twice a week for up to three weeks, in which the negative control group was treated with PBS. **(B)** Mice CT26 tumor model study was separated into 10 different groups, post-challenge treated with PBS or anti-mouse PD-1 antibody (clone 29F.1A12) are negative control group and positive control group, respectively. From group 1 to group 8 are the peptide vaccine treatment groups. Groups 1/2/5/6 were treated every three weeks, while groups 3/4/7/8 were treated every 2 weeks. ISA 720 was used in group 1/2/3/4, while ISA 51 was used in group 5/6/7/8. Nor MDP was involved in group1/3/5/7. **(C)** Immunogenicity of MVF-PD-1(92-110) peptides immunized BALB/c mice. Mice bleeds were collected weekly except the first collection was 3 weeks after primary immunization, and ELISA were used to detect antibody titers in sera; the highest dilution refer to the antibody titers as indicated in the images, for example, 12.8k indicates 1 to 12.8k or 1 to 12,800 dilution; **(D)** Antibody isotypes of BALB/c mice after immunized with MVF-PD-1(92-110) in each group. Each subtype of antibody was calculated as percentage indicated in the images. Group 1 has been published on Oncoimmunology 2020, VOL. 9, NO. 1, e1818437.

To verify and confirm the previous immunogenicity studies and antitumor activities of PD-1(92-110) (23), 6–8-week-old BALB/c mice were vaccinated with MVF-PD-1(92-110) every 2- or 3- week intervals. Peptide cancer vaccine (100 μg) mixed with ISA 720 (1:1) or ISA 51 (1:1) or nor-MDP 33 μg were used per mice. Mice were boosted with the designed doses of vaccine every 2- or 3-week intervals for a total of 3 vaccine shots. Blood was collected weekly for monitoring antibody titers. After 2 weeks of the third immunization (3Y), mice were challenged with CT26 colon carcinoma cells for 1×10^5^ cells per mouse. The tumor growths were monitored and checked daily, especially 7 days post-challenge, and the tumor size was measured with calipers **(** **Figure 1A** **)**. For the positive control group, we treated the mice with an anti-mouse PD-1 antibody (clone 29F.1A12) twice a week for up to three weeks, while the negative control group was treated with PBS. The mice CT26 tumor model study was separated into 10 different groups, post-challenge treated with PBS as the negative control group or an anti-mouse PD-1 antibody (clone 29F.1A12) as positive control group **(** **Figure 1B** **)**. Group 1 to group 8 comprised the peptide vaccine treatment groups. Groups 1/2/5/6 were treated every 3 weeks, while groups 3/4/7/8 were treated every 2 weeks. ISA 720 was used in groups 1/2/3/4, while ISA 51 was used in groups 5/6/7/8. Nor-MDP was used in groups 1/3/5/7. Mice bleed were collected weekly, except the first collection was 3 weeks after primary immunization, and ELISA was used to detect antibody titers in sera to determine the immunogenicity of MVF-PD-1(92-110) peptide-immunized BALB/c mice **(** **Figure 1C** **)**. A high-titer antibody has been established in the mice with antibody titers registering 30,000 and up to 60,000 after the third immunization. A subclass of antibody isotypes in BALB/c mice after immunization with MVF-PD-1(92-110) in each group were also analyzed as shown in **Figure 1D**. The majority of antibody isotypes were IgG1, IgG2a, and IgG2b. The percentage of each isotypes is different in the treatment groups. IgG1 ranges from 21% in group 3 to 47% in group 5. IgG2a ranges from 17% in groups 5 and 6 to 30% in group 8. IgG2b ranges from 19% in group 1 to 33% in group 3. There were very little IgM and IgA isotypes, whereas IgG3 had between 11% and 15%. There was little difference in isotype distribution between the two immune stimulants ISA720 and ISA 51. The monoclonal antibody 29F.1A12 is an IgG2a isotype antibody; here, the polyclonal antibody with different subtypes is the most advantageous compared with the single-isotype monoclonal antibody to fulfill its function. The inclusion or exclusion of nor-MDP also had little effect on immunogenicity and or in isotype distribution. Similarly, the vaccine schedule/interval of 2 weeks versus 3 weeks had very little effect on each of the parameters tested. In conclusion, the decision was made to expedite the vaccine schedule to 6 weeks versus 9 weeks and simplify the vaccine emulsion preparation by removing the nor-MDP adjuvant.

#### 3.2.2 Antitumor Efficacy in Syngeneic BALB/c Mice Challenged With CT26 Carcinoma Cell Line

**Figure 2:**
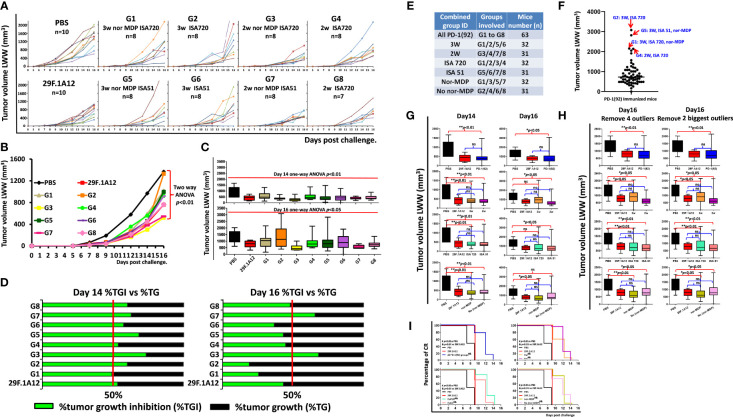
Antitumor efficacy in syngeneic BALB/c mice challenged with CT26 colon carcinoma cell line. **(A)** Individual plots of tumor growths in BALB/c mice immunized with MVF-PD-1 (92-110) vaccine, PBS as negative control, and anti-mouse PD-1 antibody (clone 29F.1A12) as positive control. As indicated, in the PBS and the mAb (29F.1A12) groups, n = 10, group 1 to group 7 n = 8, group 8 n = 7 (one mouse was lost in this group, resulting from an immunization-unrelated reason.) **(B)** Tumor burden (LWW) by days in BALB/c mice of each treatment group, and two-way ANOVA was used to analyze the whole curves of tumor growth. **(C)** Tumor burden (LWW) at day 14 and day 16 in BALB/c mice of each treatment group, and one-way ANOVA was used to analyze all 10 groups at each time point. **(D)** Percentage of tumor growth inhibition (%TGI) was used to compare the treatment groups with the PBS control group. The value was calculated by formula: %TGI=100* (MTV control − MTV test)/MTV control, both calculated at day 14 and day 16. **(E)** In this panel, we show the different combined groups: all PD-1(92); G1–G8 with n = 63; 3W are groups 1/2/5/6 with n = 32; 2W are groups 3/4/7/8 with n = 31; ISA 720 are groups 1/2/3/4 with n = 32; ISA 51 are groups 5/6/7/8 with n = 31; nor-MDP are groups 1/3/5/7 with n = 32; no nor-MDP are groups 2/4/6/8 with n = 31; **(F)** Within all the combined PD-1(92) immunized mice (n = 63), the outlier analysis was performed by using GraphPad software; four of the outliers are indicated in the image; **(G)** At Day 14 and day 16, the tumor size in different groups was analyzed by one-way ANOVA; *p < 0.05, **p < 0.01, ns, no statistical significance; **(H)** Further comparison analysis of day 16 tumor volume in different groups by removal of the 4 statistical outliers or the top two largest outliers; **(I)** the percentage of CR was defined as tumor volume equal or less than 50 mm^3^ for at least 3 consecutive measurements during the study. The log-rank (Mantel–Cox) test was used to compare the percentage of CR in multiple groups: #*p *< 0.01 versus PBS and *p *< 0.01 versus 29F.1A12.

We have previously established the rate of tumor growth by vaccination with various peptide vaccines and treatments with the anti-mouse PD-1 antibody (clone 29F.1A12). Each group of mice immunized with MVF-PD-1(92-110) peptide was compared with PBS negative control and a positive control group with anti-mouse PD-1 antibody (clone 29F.1A12); the tumor volume was calculated by LWW (**Figures 2A, B**) for individual mouse and the different treatment groups, respectively. The number mice in each group as indicated, PBS and mAb 29F.1A12 n = 10, group 1 to group 7 n = 8, and group 8 n = 7. The results showed that in the majority of the mice, the tumor size has been significantly inhibited by the peptide vaccine and anti-mouse PD-1 antibody (clone 29F.1A12) versus with the PBS group with a *p*-value less than 0.01 by the two-way ANOVA of a whole-curve comparison. At day 14 and day 16, the one-way ANOVA indicated *p*<0.01 and *p*<0.05, respectively, of a 10-group comparison (**Figure 2C**). The percentage of tumor growth inhibition analysis (**Figure 2D**) at day 14 and day 16 showed about 50% or above tumor growth inhibition of all treatment groups compared with the PBS group, especially at day 14. For day 16%TGI, due to few mice with limited tumor control ability result in G1 and G2 seems with relatively lower %TGI, while G3 and G7 mice showed higher tumor growth inhibition ability.

In order to better understand the 2-week vs. 3-week interval, ISA 720 vs. ISA 51, and nor-MDP vs. no nor-MDP strategy to assess the best option for the human clinical trial, further analysis was performed by combining some groups together (see **Figure 2E**). Within all the 63 PD-1(92)-immunized mice, a number of mice had a larger tumor size from day 14 to day 16. The outliers, 1 mouse in G2 (3W ISA 720), 1 mouse in G5 (3W, ISA 51, nor-MDP), 1 mouse in G1 (3W, ISA 720, nor-MDP), and 1 mouse in G4 (2W, ISA 720), were identified by GraphPad software outlier analysis (**Figure 2F**). We found that within all the combined groups with significant difference versus the PBS group showed no difference with the mAb group 29F.1A12 group at day 14. Several mice at day 16 showed a larger tumor volume, resulting in no significant difference versus the PBS group, for example, 3W vs. PBS, ISA 720 vs. PBS, and no nor-MDP vs. PBS (**Figure 2G**). However, upon further analysis by removing the four statistical outliers or the two largest outliers in the combined groups, all the groups are significantly different with PBS groups (**Figure 2H**). Notably, the percentage of complete response indicated that all the combined immunized groups showed a significant difference compared with PBS and mAb (29F.1A12) group (**Figure 2I**). This suggested that the mice immunized with the PD-1(92) vaccine showed a higher percentage of complete response. A limitation in the present studies is that we did not track the live chart as the aims and goals of the study were to verify the immunogenicity of immunized mice in the different categories such as a 3-week versus 2-week interval, the removal of nor-MDP, and the use of ISA720 versus ISA51 to provide clinical information for the IND application to the FDA.

Overall, the peptide-immunized mice showed good tumor inhibition comparable to monoclonal antibody-treated mice. There was no difference in this study between the peptide vaccine- treated group and the anti-mouse PD-1 antibody (clone 29F.1A12)-treated group. Additionally, we did not find a difference between 2-week interval immunization and 3-week interval immunization strategies.

Altogether, our CT26/BALB/c tumor model indicated that there was no significant difference between 2-week and 3-week interval immunization strategies after mice were challenged with the CT26 colon carcinoma tumor cells. These two different immunization methods showed a similar tumor inhibition ability by the novel peptide vaccine, PD1-Vaxx. The only noticed difference between 2-week and 3-week interval immunization is the immunogenicity response before the third (3Y) immunization. Before 3Y immunization, 2-week-interval immunized mice showed relatively lower antibody titers compared with the same point in mice immunized every 3 weeks. However, after the 3Y, a super high immune response in mice was obtained with either independent immunization strategy. There was no significant difference between 2-week interval and 3-week interval, ISA 720 and ISA 51, or with or without nor-MDP, respectively.

#### 3.2.3 MVF-PD-1 (92-110) Peptide Shows High Immunogenicity in Beagle Dogs

**Figure 3:**
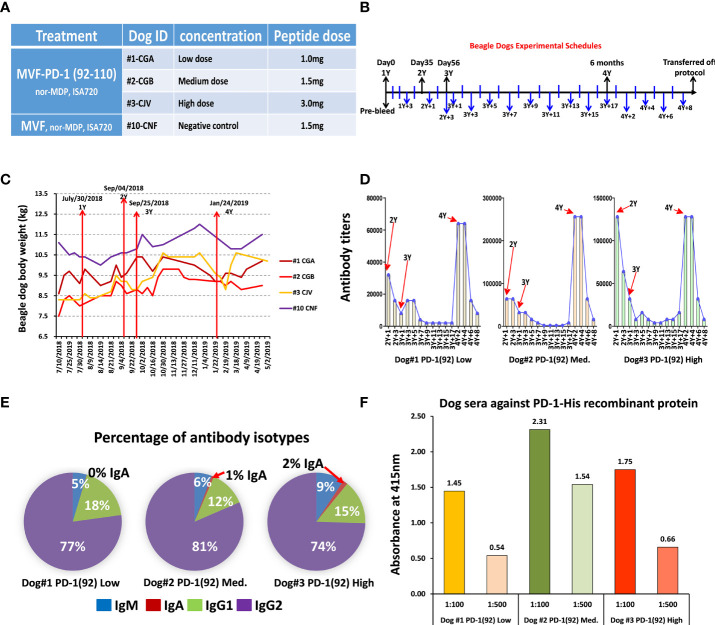
Immunogenicity and safety in Beagle dogs. **(A)** There were 4 beagle dogs involved in this study. Dog #1, #2, and #3 were treated with single MVF-PD-1(92-110) nor-MDP and ISA 720 with different doses. Dog #10 was a negative control; **(B)** Dogs were immunized 4 times, which point to 1Y, 2Y, 3Y, and 4Y at day 0, day 35, day 56, and 6 months after primary immunization, respectively. Blood was collected every two weeks except the first collection was 3 weeks after the primary immunization and 1 week after 2Y and 3Y immunization. The sera were used to monitor the antibody titers. All the beagle dogs were transferred out of protocol after this preclinical study. **(C)** The plot of dogs’ body weight during the experimental period. **(D)** Immunogenicity of peptide-based vaccines in beagle dogs. ELISA were used to detect antibody titers in sera. **(E)** Isotypes IgM, IgA, IgG1, and IgG2 in canine were detected after being immunized with MVF-PD-1(92) peptide vaccine. **(F)** Dogs’ sera antibody activity with human recombinant protein PD-1 His.

This study was designed to monitor the kinetics of how quickly the antibody levels to MVF-PD-1(92-110) diminished after the dosing was stopped. The recall of an antibody response was also tested after re-challenge. Four beagle dogs were used in this study of MVF-PD-1(92-110) peptide cancer vaccine. Dogs #1, #2, and #3 were treated with MVF-PD-1(92-110) emulsified with nor-MDP and ISA 720 using different doses **(** **Figure 3A** **)**. Dog#1 was treated with 1.0 mg low-dose peptide, dog#2 was treated with 1.5 mg medium-dose peptide, and dog #3 was treated with 3.0 mg high-dose peptide. The negative control dog #10 was only treated with the MVF peptide emulsified with nor-MDP and Montanide ISA720. Dogs were immunized 4 times designated as 1Y, 2Y, 3Y, and 4Y at Day 0, Day 35, Day 56, and 6 months after primary immunization, respectively. Blood was collected every 2 weeks, except for the first collection, which was 3 weeks after the primary immunization and 1 week after 2Y and 3Y immunization **(** **Figure 3B** **)**. The sera were used to monitor the antibody titers. We monitored the beagle dogs’ body weight closely during the immunization period **(** **Figure 3C** **)**. As shown, all the dogs have gained some weight by the end of the experiment compared with the initial weight.

ELISA was used to detect antibody titers in sera. For the dogs treated with MVF-PD-1(92-110) peptide, the vaccine elicited a high titer antibody; especially after 4Y immunization, the antibody titers are equivalent or even much higher than before **(** **Figure 3D** **)**. This study was designed to monitor the kinetics of how quickly the antibody levels to MVF-PD-1 (92-110) (APi2568) diminished after the dosing was stopped. The recall of an antibody response was also tested after a re-challenge. As shown in **Figure 3D**, this exploratory study demonstrated antibody titers dropped to very low levels in the absence of dosing but could be recovered if the dog was re-challenged with vaccine.

In order to investigate the diversity of the antibody produced by immunized dogs, isotypes IgM, IgA, IgG1, and IgG2 in dogs were detected after being immunized with the peptide vaccine **(** **Figure 3E** **)**. In MVF-PD-1(92-110) vaccine-treated dogs, the majority of isotype is IgG2, which ranged from 74% to 81%, the IgG1 range from 12% to 18%, IgM from 5% to 9%, and IgA less than 2%. Peptide sera from dogs #1, 2, and 3 were used to detect the antibody activity against human recombinant protein PD-1. Dog #2 with medium dose had higher reactivity than the low-dose dog #1 or highest-dose dog #3, indicating that the medium dose is likely the optimum dose in dogs **(** **Figure 3F** **)**. In conclusion, as shown in **Figure 3E**, there was a strong bias toward the antibodies of the IgG2 type.

Collectively, all dogs responded to immunization with PD1-Vaxx eliciting a robust polyclonal antibody response. Titers reduced to baseline levels once dosing was ceased but could be recalled if the dogs were boosted with vaccine at 6 months. No lesions or adverse observations were noted when the dogs were boosted at 6 months.

#### 3.2.4 Conclusions of Mice and Dog Studies

Based on these results in mice and dogs, the emulsion formulation of MVF-PD-1(92-110) considered optimal for further development consisted of a 1:1 mixture of an aqueous solution of MVF-PD-1(92-110) and Montanide ISA 720 VG solution; this formulation was designated PD1-Vaxx (emulsion of the proposed drug product, IMU-201 with Montanide ISA 720 VG).

In view of the 100% homology between human and cynomolgus monkeys for the target PD-1 (**Table 1**), the cynomolgus monkey was proposed as the species for nonclinical Good Laboratory Practice (GLP)-compliant toxicology studies.

**Table 1:** The homology of PD-1 peptide sequence in species of human, cynomolgus, rhesus, mouse, canine, and rat.

Species	PD-1 Peptide Sequence	Homology
Human PD-1	**GAISLAPKAQIKESLRAEL**	100%
Cynomolgus PD-1	**GAISLAPKAQIKESLRAEL**	100%
Rhesus PD-1	**GAISLAPKAQIKESLRAEL**	100%
Mouse PD-1	**GAISLHPKAKIEESPGAEL**	74%
Canine PD-1	**GAIYLPPNTQINESPRAEL**	68%
Rat PD-1	**GAISLPPKAQIKESPGAEL**	84%

Colored letters indicate the difference of the amino acid with the human sequence.

#### 3.2.5 Anti-APi2568 Antibody Titers in Monkey Serum After Capture by RhPD-1

The non-human primates were approximately 2−4 years old and weighed between 2.2 and 3.1 kg at the initiation of dosing. Two sets (#00213515 and #00213519) of non-human primate cynomolgus monkeys study were performed at Charles River Laboratories (Ashland, OH, USA). All the cynomolgus monkeys were immunized with MVF-PD-1(92) (PD1-Vaxx, APi2568), with a total of 40 non-human primates. The immunization strategies were done on a 2-week interval based on our previous preclinical BALB/c mice model studies.

**Figure 4:**
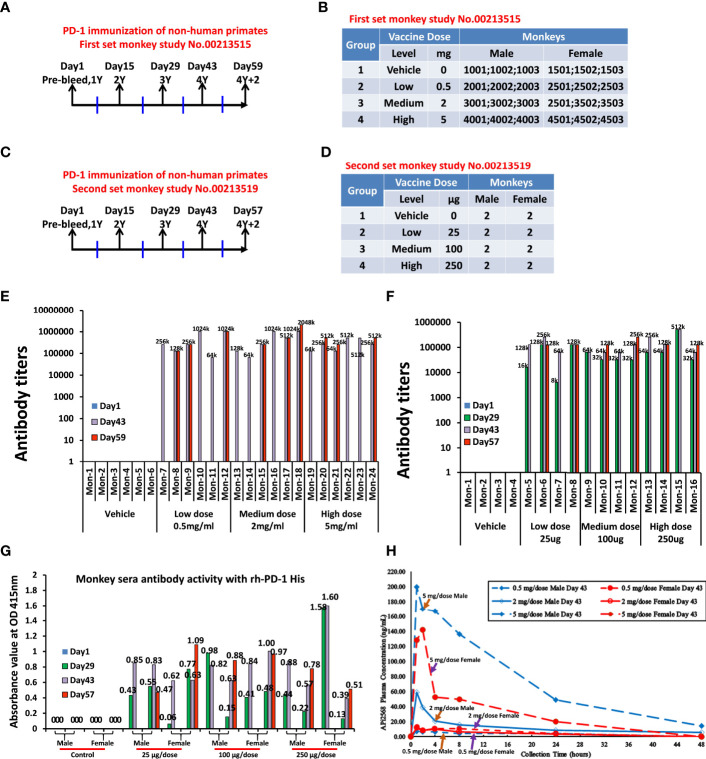
Non-human primates’ study, anti-APi2568 antibody in monkey serum activity with rh-PD-1 His and Half-life of APi2568 (MVF-PD-1(92)) in the monkey plasma. **(A)** First set of monkey study No.00213515, non-human primates, cynomolgus monkeys, were used in this study. The monkeys were immunized three times, which point 1Y, 2Y, 3Y and 4Y with 2-weeks interval. The bleeds were collected at pre, 2Y+2/3Y, 3Y+2 and 4Y and 4Y+2. **(B)** There are four groups and 6 monkeys in No.00213515 of each group. Group 1 is the control group, which was treated with only ISA 720 and water. Group 2, group 3, and group 4 are peptide treatment groups, which were treated with low 0.5 mg/dose, medium 2 mg/dose, and high 5 mg/dose in No.00213515; **(C)** on the second set of non-human primate study No.00213519, 16 of cynomolgus monkeys were used in this study. The monkeys were immunized three times, which point 1Y, 2Y, 3Y, and 4Y with 2-week intervals. The bleed was collected at pre, 2Y+2/3Y, 3Y+2 and 4Y and 4Y+2. **(D)** There are four groups and 4 monkeys in No.00213519 of each group. Group 1 is the control group, which was treated with only ISA 720 and water. Group 2, group 3, and group 4 are peptide treatment groups, which were treated with low 25 μg/dose, medium 100 μg/dose, and high 250 μg/dose of peptide vaccine in No.00213519; **(E, F)** Immunogenicity of peptide-based vaccines in non-human primates. ELISAs were used to detect antibody titers in sera of both monkey study No.00213515 and No.00213519, respectively; the numbers indicated the highest dilution of sera, titers; **(G)** Absorbance value at OD 415 nm of monkey serum immunized with APi2568 [MVF-PD-1(92)] peptide vaccine against human recombinant protein PD-1 activity. The 1:100 diluted sera were used. **(H)** Half-life of APi2568 (MVF-PD-1(92) peptide vaccine in the monkey plasma at day 43 after immunization, which indicated that the relatively higher dose has relatively longer half-life in the monkey plasma, both in male and female.

In the non-human primate study (#00213515), 24 non-human primates, cynomolgus monkeys, were used. The monkeys were immunized four times (1Y, 2Y, 3Y, and 4Y at days 1, 15, 29, and 43, respectively). Sera were collected at day 1 (pre), day 43, and day 59 (**Figure 4A**). There were four groups and 6 monkeys in each group. Group 1 was the control group, which was treated with only ISA 720 and ddwater. Groups 2, 3, and 4 were peptide PD-1Vaxx treatment groups and were treated with low-dose 0.5 mg/dose, medium-dose 2 mg/ml, or high-dose 5 mg/ml peptide vaccine, respectively (**Figure 4B**), emulsified in ISA 720.

In the second set of non-human primate study (#00213519), 16 non-human primates, cynomolgus monkeys, were used. The monkeys were immunized three times at 2-week intervals represented by 1Y, 2Y, 3Y, and 4Y at days 1, 15, 29, and 43, respectively.The bleed were collected at day 1 (pre), day 29 (2Y+2/3Y), day 43 (3Y+2 and 4Y), and day 57 (4Y+2) (**Figure 4C**). There were four groups with 4 monkeys (2 males and 2 females) in each group. Group 1 was the control group, which was treated with only ISA 720 and ddwater. Group 2, group 3, and group 4 were peptide treatment groups, which were treated with low-dose 25 μg/dose, medium-dose 100 μg/dose or high-dose 250 μg/dose of PD1-Vaxx peptide vaccine, respectively (**Figure 4D**). The Immunogenicity of peptide-based vaccines in non-human primates was detected by using ELISA to detect antibody titers in the sera of both non-human primate studies (**Figures 4E, F**). The relative high antibody responses have been established in each individual cynomolgus monkey.

Using the serum collected from cynomolgus monkeys in the GLP-toxicology study, where monkeys were administered IMU-201 (as PD1-Vaxx) at APi2568 doses of 25, 100, and 250 µg on days 1, 15, 29, and 43 (4 total doses) and serum samples collected on days 1, 29, 43, and 57 (after a 2-week recovery period) were evaluated for anti-APi2568 antibody titers. The anti-APi2568 antibody titers were high and were relatively uniform across sample collection days and between the APi2568 dose groups. However, these antibody titers were determined using APi2568 to capture the formed antibodies and did not determine if the formed antibodies would recognize PD-1. In a separate experiment, these monkey serum samples were tested using recombinant human PD-1 (rhPD-1) as the capture agent. **Figure 4G** shows the individual monkey anti-APi2568 antibody in sera on days 29, 43, and 57 activities against human recombinant PD-1 protein. These antibody results indicated that some of the anti-APi2568 antibody formed in monkeys recognized rhPD-1, suggesting that the anti-APi2568 antibodies formed in humans after vaccination with PD1-Vaxx would recognize PD-1 expressed on immune cells. See **Table 2** for summary of mouse, dog, and non-human primate experiments.

**Table 2:** Summary of mouse, dog, and non-human primate experiments.

Species	Number	Immunization	Intervals	Adjuvants	Nor-MDP	Tumor	Results
**Mouse**	**83**	**PD-1(92) or control**	**2 weeks** **and 3 weeks**	**ISA 720 and ISA51**	**With or no**	**CT26**	**High immunogenicity (** **Figure 1C** **);**
							**PD-1(92) significant less tumor burden vs. PBS, no difference vs. 29F.1A12 (** **Figures 2G, H** **);**
							**2-week vs. 3-week no difference (** **Figures 2G-I** **);**
							**ISA 720 vs ISA 51 no difference (** **Figures 2G-I** **);**
							**No nor-MDP vs nor-MDP no difference (** **Figures 2G-I** **);**
**Dog**	**4**	**PD-1(92) or control**	**Based on 3 weeks**	**ISA 720**	**With**	**No**	**Peptide vaccine is safe and with high immunogenicity (** **Figure 3D** **), and high activity against recombinant PD-1 protein (** **Figure 3F** **);**
**Monkey 1st set**	**24**	**PD-1(92) or control**	**2 weeks**	**ISA 720**	**No**	**No**	**Peptide vaccine is safe and with high immunogenicity (** **Figures 4E, F** **); antibody with high binding ability against rh-PD-1 (** **Figure 4G** **);**
**Monkey 2nd set**	**16**	**PD-1(92) or control**	**2 weeks**	**ISA 720**	**No**	**No**	

### 3.3 Pharmacokinetics

As part of a GLP-compliant, toxicology, toxicokinetic (TK), and immunogenicity study (Charles River Laboratories, Ashland, Ohio, OH, United States), day 1 and day 43 systemic exposure (or TK) profiles of APi2568 [filled and finished(UI Pharmaceuticals, Iowa City,I A ) MVF-PD-1(92-110)] in male and female cynomolgus monkeys were determined after the APi2568 doses of 0.5, 2, and 5 mg/dose were administered IM as PD1-Vaxx (see **Table 3**).

**Table 3:** Mean toxicokinetic parameters for Api2568 in male and female monkeys on day 1 and day 43.

Gender	Dose (mg/dose)	Day	C_max_ (ng/ml)	C_max_/D	T_max_ (h)	T_last_ (h)	T_1/2_ (h)	AUC_(0-t)_ (ng·h/ml)	AUC_(0-t)_/D
Male	0.5	1	NA	NA	NA	NA	NC	NC	NC
		43	7.21	7.21	1	8	NC	62.3	125
	2	1	NA	NA	NA	NA	NC	NC	NC
		43	59.3	59.3	1	24	13.1	494	247
	5	1	10.1	2.01	2	24	NC	151	30.1
		43	202	40.5	1	48	12.7	3,460	693
Female	0.5	1	NA	NA	NA	NA	NC	NC	NC
		43	8.81	17.6	2	4	NC	223	446
	2	1	NA	NA	NA	NA	NC	NC	NC
		43	13.8	6.92	4	8	NC	94.5	47.3
	5	1	4.77	0.954	1	4	NC	18.9	3.79
		43	160	31.9	1	34	19.6	1,330	266

Units for Cmax/D and AUC(0-t)/D are (ng/ml)/(mg/dose) and (ngh/ml)/(mg/dose), respectively. H, hour; IMC, not calculable; NA, not applicable; C_max_, maximum observed plasma concentration; T_max_, time when Cmax was observed; AUC, area under the plasma concentration time curve.

For the determination of TK parameters, blood samples were collected at various times after dosing analyzed using a validated UHPLC-MS/MS (ultra-high-performance liquid chromatography with tandem mass spectrometry) method, for the quantification of APi2568 in monkey plasma.

Following the IM administration of APi2568 at 0.5, 2, and 5 mg/dose to male and female monkeys, measurable plasma APi2568 concentrations on day 1 were insufficient to calculate AUC values, except in 1 monkey/sex at 5 mg/dose. After APi2568 repeated dosing (every 14 days for 4 doses), all monkeys were systemically exposed to APi2568 by day 43 with exposure in female monkeys generally lower than that observed in male monkeys.

**Figure 4H** presents the mean (generally n = 3) APi2568 plasma concentration half-life time profiles for each APi2568 dose level on day 43 from which we can see that the relatively higher dose would result in the relatively longer half-life in the plasma both in male and female monkeys. Notably, we observed that the APi2568 has a longer half-life in the male monkey than female, especially in high dose and medium dose as shown in the **Figure 4H**.

On Day 43, APi2568 plasma concentrations remained near (≤6-fold) the LLOQ (lower limit of quantification) in the 0.5 mg/dose group (both sexes) and in the 2 mg/dose female monkeys. After reaching C_max_ at 1−4 h postdose, APi2568 plasma concentrations generally decreased steadily through the last measurable time point (approximately 48 h after dosing) in both sexes at all APi2568 dose levels. The extent (C_max_) and duration (AUC_(0-t)_) of exposure to APi2568 on day 43 increased as the APi2568 dose level increased from 0.5 to 5 mg/dose in male monkeys and generally in female monkeys, except that a single female monkey at the 2 mg/dose level had significantly lower exposure than the other 2 female monkeys at this dose level, resulting in a lower mean AUC_(0-t)_. The increase in APi2568 exposure (based on mean AUC_(0-t)_/D values) was greater than dose-proportional from 0.5 to 5 mg/dose in male monkeys and could not be determined in female monkeys due to the inconsistent APi2568 plasma concentrations in the 0.5 and 2 mg/dose groups. APi2568 T_1/2_ values were calculable only on day 43 for the 2 and 5 mg/dose male monkeys and a single 5 mg/dose female monkey and had a mean value of approximately 13 h. The accumulation of APi2568 after the repeat-dose administration of PD1-Vaxx was apparent since more plasma samples on day 43 had measurable and higher APi2568 concentrations compared to those in plasma samples on Day 1 at the same collection time. The accumulation ratios (R_A_) for the single male and female monkey at the 5 mg/dose with TK profiles on both days 1 and 43 were 16.3 and 121, respectively. These TK results (**Table 3** **)** for APi2568 indicated that male and female monkeys were exposed to APi2568 on Day 1 (5 mg/dose only) and on Day 43 (all dose levels) with male monkeys having somewhat greater exposure compared to that in female monkeys and that some APi2568 accumulation was present after the repeat-dose IM administration of PD1-Vaxx.

### 3.4 Toxicology

Repeat-dose toxicology and immunogenicity evaluations on APi2568 (IMU-201-administered IM as PD1-Vaxx) have been conducted, and the results from a pharmacology immunogenicity study conducted in beagle dogs and from 2 GLP-compliant toxicology studies conducted in male and female cynomolgus monkeys are summarized below.

#### 3.4.1 Beagle Dog Study

a) Beagle dogs were immunized with MVF-PD-1 (92-110) (3 dogs with 1 dog each at the doses of 1, 1.5, and 3 mg/dose).
b) All dogs administered with MVF-PD-1 (92-110) had high polyclonal anti-MVF-PD-1 (92-110) antibody titers. Two out of three dogs administered with MVF-PD-1 (92-110) had an adverse local reaction (i.e., lesions at or near the IM injection site) to the vaccine; after the dosing ceased, these lesions resolved within a few weeks. These injection site lesions were mostly attributed to the high volume of Montanide ISA 720 VG used in this study.

#### 3.4.2 First Set of Monkey Study

a) The findings of adverse swelling, ulceration, and/or abscess formation at or near the IM site of dose administration were observed for monkeys in the 0.5, 2, and 5 mg/dose groups. These observations were first noted beginning after the second or third dose and were more frequent in female monkeys compared to male monkeys.
b) After the recovery period, all APi2568-related clinical observations had either recovered or were in the process of recovering, with the exception of swollen hind limbs. APi2568-related changes in hematology, coagulation, and serum chemistry parameters were noted at ≥0.5 mg/dose and occasionally correlated with the microscopic finding of inflammation at the IM injection sites.
c) With a few exceptions, all clinical pathology parameter changes recovered. The APi2568-related macroscopic and microscopic findings involving the IM injections sites were noted at each APi2568 dose level evaluated. After the 14-day recovery period, a complete recovery of the microscopic findings was noted in the epidermis, a partial recovery of some inflammatory findings, and no recovery of the cavity and hemorrhage findings.
d) Clinical observations and histopathological findings involving the IM injection sites were noted in the vehicle control group monkeys at a lower frequency and reduced severity relative to that in the APi2568-treated groups, suggesting a contribution of the vehicle to the observations and findings with an exacerbation by APi2568.
e) Based on these results, and primarily considering the adverse clinical findings observed at the IM sites at 0.5, 2, and 5 mg/dose APi2568 dose levels evaluated, a no-observed-adverse-effect level (NOAEL) for APi2568 administered IM as PD1-Vaxx to male and female cynomolgus monkeys could not be determined for this study.

#### 3.4.3 Second Set of Monkey Study

a) The IM injection of PD1-Vaxx to male and female cynomolgus monkeys at APi2568 dose levels of 25, 100, and 250 µg/dose administered on 4 separate occasions at 2-week intervals resulted in adverse necrosis (the loss of normal tissue and abundant cellular debris) in the biceps femoris IM injection site of a single 250 µg/dose group female monkey (**Table 4**).
b) APi2568-related clinical, injection site, and gross necropsy findings consistent with swelling were also noted for this female monkey.
c) Non-adverse APi2568-related neutrophilic inflammation was noted in some monkeys in both the treated and control groups, indicating at least a partial contribution of the adjuvant or dose administration procedure to the finding. However, this finding was observed at a greater severity in APi2568-treated male and female monkeys.
d) Some non-adverse adjuvant-related microscopic findings were noted in some monkeys in all APi2568 treatment groups and the control group.
e) Generally, anti-APi2568 antibody titers were comparable for male and female monkeys at the APi2568 dose levels of 25, 100, and 250 µg/dose. A slight increase in anti-APi2568 antibody titers was observed between day 29 and day 43, with no apparent reduction in titers by day 59.

**Table 4:** Second-set monkey study (APi2568) IMU-201 administered IM as PD1-Vaxx.

Gender	Dose (µg/dose)	Immunization	Aim	Findings
**Male** **and** **female**	**0; 25; 100; 250;**	**Day 1; day 15; day 29; day 43;** **with a 14-day** **recovery** **period.**	**1: Lower APJ2568 doses were used to determine if the adverse IM injection site effects observed in the first study could be prevented by reducing the APJ2568 doses** **2: To determine if these lower APJ2568 doses were able to produce the desired immunogenic response of generating significant titers of anti-APi2568 antibodies**	**1: All monkeys survived to the scheduled necropsy.** **2: No APi2568-related adverse effects were noted on body weight, food consumption, body temperature, clinical pathology parameters, or organ weight.** **3: No TK evaluations or cardiovascular parameter assessments were made during this study.**

A single female monkey in the 250 ng/dose group had an APi2568-related mass present at the IM injection site on the right hindlimb 7 days following the third dose. This observed mass remained unchanged until the day of scheduled terminal euthanasia for this monkey, correlated with the APi2568-related injection site observation of very slight edema, was associated with the APi2568-related gross necropsy finding of swelling in the right biceps femoris IM-injected site, and correlated with the APi2568-related histopathologic finding of moderate neutrophilic inflammation. In addition, a microscopically observed, APi2568-related necrosis in the right biceps femoris IM site of this female monkey was characterized by the loss of normal tissue and abundant cellular debris and this finding was considered to be adverse. No other APi2568-related gross necropsy findings were noted at the terminal or recovery euthanasia.

Based on these results, the NOAEL was 100 µg/dose for this study on APi2568-administered IM as PD1-Vaxx to male and female cynomolgus monkeys.

### 3.5 No Autoimmune Symptoms Observed After the Immunization

The common autoimmune symptoms are identified as follows: skin rashes, low-grade fever, achy muscles, fatigue, hair loss, trouble concentrating, and so on. During our mice study, we did not notice any autoimmune symptoms, the mice showed no signs of scruffiness, lesions, or lethargy, and all of them were active during the study. For the dogs, the high volume of ISA 720 caused a lesion near the injection site that might result in mild swelling, but the symptoms were resolved weeks after. The immunized monkeys had some swelling at or near the site of IM injection, especially after the second or third dose and more frequent in females than males. In all the clinical observations, these animals either had recovered or were in the process of recovering. Despite these adverse clinical findings, all of them were active and in good condition throughout the project. Overall, there were no observable signs or uncontrolled autoimmune symptoms.

## 4 Discussion

We are developing APi2568 [clinical-grade MVF-PD-1(92-110)], the drug substance in the drug product IMU-201 administered IM as PD1-Vaxx, to treat cancers that overexpress programmed cell death ligand 1 (PD-L1), such as non-small cell lung cancer (NSCLC), melanoma, renal cancer, and colorectal cancer. IMU-201 is administered IM to cancer patients as an emulsion formulation, designated PD1-Vaxx, which is made by combining equal volumes of IMU-201 (an aqueous solution of APi2568) and a solution of Montanide ISA 720 VG followed by emulsification. The objective of immunization with PD1-Vaxx is to induce the production of anti-PD-1 antibodies in cancer patients using a peptide epitope designed to stimulate polyclonal antibodies against PD-1 (programmed cell death protein 1). This vaccination approach is based on the use of a chimeric conformational B-cell epitope peptide incorporating a “promiscuous” T-cell epitope that affords the possibility of generating an enduring immune response, eliciting protein-reactive, high-affinity anti-PD-1 antibodies that act as antagonists to receptor signaling that inhibits T-cell responses to tumors. As summarized in this manuscript, the effective induction of an antibody response to MVF-PD-1 (92-110) has been established in a variety of tumor models in mice and other animal species such as beagle dogs and cynomolgus monkeys. The induction of autoimmune responses has not been observed in any of these animal species. We have completed the nonclinical toxicology and immunogenicity evaluations of IMU-201 (administered IM as PD1-Vaxx) in male and female cynomolgus monkeys that have a high degree (>90%) of PD-1 sequence homology to humans, including 100% homology for the 19 PD-1 amino acids in MVF-PD-1 (92-110), prior to initiating clinical trials on IMU-201 in cancer patients.

The nonclinical pharmacology assessments on MVF-PD-1 (92-110) consisted of experiments to select the candidate B-cell epitopes expressed on the surface of PD-1 for development and then to evaluate that candidate (designated MVF-PD-1 (92-110) and later, the drug substance or APi2568) for pharmacology activity. MVF-PD-1 (92-110), which is a chimeric construct with a promiscuous T-cell helper epitope derived from the MVF protein (amino acids 288-302), was shown to recognize and bind both rhPD-L1 and nivolumab. *In vivo* studies in Balb/c mice showed that MVF-PD-1 (92-110) was emulsified with nor-MDP and Montanide ISA 720 VG and injected 3 times once every 3 weeks to immunize the mice produced high titers of anti-MVF-PD-1 (92-110) antibodies and was able to reduce the tumor growth of CT26 tumor cells engrafted 10 days after the last MVF-PD-1 (92-110) dose. Other *in vivo* studies in mice showed that the nor-MDP excipient was not necessary and that Montanide ISA 720 VG produced a lower ratio of IgG1 antibodies (known to result in increased ADCC) compared to Montanide ISA 51 VG. In beagle dogs immunized with an emulsion formulation of MVF-PD-1 (92-110), the majority isotype generated was IgG2 with a significantly lower generation of IgG1 and IgM and a little production of IgA. During this study, a number of dogs exhibited an adverse reaction with lesions forming in the area of the injection site. Based on these results in mice and dogs, the emulsion formulation of the clinical grade of APi2568 considered optimal for further development consisted of a 1:1 mixture of an aqueous solution of APi2568 (drug product, IMU-201) and Montanide ISA 720 VG, which was designated as PD1-Vaxx.

The completed pharmacology effectiveness studies on APi2568 indicate that APi2568 administered alone can reduce the growth of the CT26 tumor cells implanted into mice after immunization. APi2568 has been shown to produce relatively high anti-APi2568 antibody titers in mice, dogs, and monkeys. In monkeys, the antibody titers were relatively uniform across the APi2568 dose levels evaluated, were generally higher in female monkeys compared to those in male monkeys, and were not substantially reduced 2 weeks after the final dose of APi2568 was administered. After 4 doses of APi2568 at 25 µg/dose, the monkey anti-APi2568 antibody titers were similar to those produced by the 100 µg/dose and 250 µg/dose, suggesting that APi2568 is highly immunogenic in this species and will most likely be immunogenic in humans since the 19 PD-1 amino acids in API2568 have 100% homology between humans and monkeys (see **Table 2**). In a separate experiment, some of the anti-APi2568 antibodies present in the monkey serum were captured by rhPD-1, suggesting that the anti-APi2568 antibodies formed in humans after the administration of IMU-201 as PD1-Vaxx should act as anti-PD1 antibodies to block PD-1 on immune cells.

The systemic circulation exposure profile of APi2568 has been determined in male and female monkeys. After the first IM dose of APi2568, plasma concentrations were insufficient to evaluate the extent and duration of exposure except in 1 monkey/sex in the high-dose group (5 mg/dose). After 4 doses of APi2568, most monkeys at each APi2568 dose level had some plasma samples with quantifiable APi2568 and selected pharmacokinetic parameters could be determined for many monkeys in the 2 mg/dose and 5 mg/dose groups. These assessments indicated that female monkeys were generally exposed to less APi2568 than male monkeys, that APi2568 was cleared from systemic circulation by 48 h after dosing, and that some accumulation of APi2568 was possible after repeated IM dose administration.

Toxicology findings on APi2568 (administered IM as PD1-Vaxx) in male and female monkeys suggest that APi2568 doses >0.5 mg/dose can cause significant and adverse local tolerance effects at or near the IM injection site. These adverse effects included swelling, ulceration, and/or abscess formation with very slight-to-slight edema and erythema; were first noted after the second or third PD1-Vaxx dose; were generally more frequent and more severe in female monkeys compared to male monkeys; and had clinical pathology and histopathological correlates. Vehicle control monkeys had similar findings but at a lower frequency and reduced severity, suggesting a possible contribution of the vehicle (containing Montanide ISA 720 VG) with an exacerbation by APi2568. When the dose of IM-administered APi2568 was reduced, these adverse local tolerance effects were not observed at APi2568 doses of <100 µg/dose, and only a single female monkey in the 250 µg/dose group had an adverse local tolerance finding of an APi2568-related mass present at the IM injection site on the right hindlimb 7 days following the third dose. These findings indicated that APi2568 at 100 µg/dose has an acceptable toxicology or safety profile in male and female monkeys.

## 5 Conclusion

The anti-PD1 B-cell epitope vaccine eliciting a polyclonal antibody response has several advantages over mAb treatments. The advantages include the induction of memory B- and T-cell responses without causing potentially serious hypersensitivity reactions, infusion reactions, and immune complex-mediated diseases that inflict substantial mental, biological, and financial burdens upon the cancer patients. Additional benefits of the peptide vaccine approach include the ease and rapid synthesis, safety, lack of toxicity, and cost-effectiveness (24). Based on the findings of the completed pharmacology, immunogenicity, pharmacokinetic, and toxicology studies, active immunization with MVF-PD-1(92-110) peptide vaccine (**Table 1**) 1) is effective in lowering the tumor burden in murine models of cancer; 2) is highly immunogenic in mice, dogs, and monkeys with relatively high anti-PD-1 antibody titers present in monkeys at MVF-PD-1(92-110) doses as low as 25 µg/dose; 3) has an acceptable systemic exposure profile with some possible accumulation after repeated IM administration; 4) has an acceptable toxicology and safety profile after repeat-dose IM administration at MVF-PD-1(92-110) doses of less than 100 µg/dose; and 5) exhibited the conservation of dose across animal species despite differences in body surface area (BSA), indicating that translation to the human effective dose (HED) would not require a BSA-based conversion factor typically required for monoclonal antibodies and small molecules (25). These observations suggest that clinical trials on peptide-based vaccine MVF-PD-1(92-110) using an active immunization approach consisting of a 2-week priming dose regimen with a maximum recommended starting dose (MRSD) of 10 µg/dose increasing in ½ log increments to a high dose of 100 µg/dose should not produce significant adverse effects or toxicity, including IM injection site intolerability.

## Data Availability Statement

The raw data supporting the conclusions of this article will be made available by the authors, without undue reservation. Further inquiries can be directed to the corresponding author: Kaumaya.1@osu.edu.

## Ethics Statement

The animal study was reviewed and approved by Ohio State University Institutional Animals Care and Use Committee and detailed in the accepted protocol (2009A0013-R2:2/5/2015-2/5/2018; 2009A0013-R3:1/18/2018-1/18/2021;2009A0013-R4 and 11/9/2021-11/9/2024).

## Author Contributions

PK contributed to conception and design, supervised and managed the data generation and data analysis, and edited and wrote the sections of the manuscript including final edits. LG wrote the initial draft and contributed to the generation of the data in mice and dogs, statistical analysis and editing, and the preparation of figures. JO performed the mice and dogs studies and generated the data. AJG and NJE contributed to the design and preparation of the IND documents for FDA approval, review and editing of all versions of the manuscript. PK, LG, JO, AJG, and NJE contributed to review drafts and editing all versions of the draft. All authors gave a final acceptance of the manuscript.

## Funding

This study received funding from Imugene Ltd [OSU 900600] to PK. Imugene was not involved in the study design, collection, analysis, interpretation of data, the writing of this article, or the decision to submit it for publication. Imugene funded the studies in the non-human primate studies contracted to Charles River laboratories and provided the summaries.

## Conflict of Interest

AG and NE are employed by Imugene Ltd. PK is a consultant to Imugene, Ltd.

The remaining authors declare that the research was conducted in the absence of any commercial or financial relationships that could be construed as a potential conflict of interest.

## Publisher’s Note

All claims expressed in this article are solely those of the authors and do not necessarily represent those of their affiliated organizations, or those of the publisher, the editors and the reviewers. Any product that may be evaluated in this article, or claim that may be made by its manufacturer, is not guaranteed or endorsed by the publisher.
